# Effect of sainfoin (*Onobrychis viciifolia* Scop.) seed‐based diet on rats: A comprehensive evaluation of hemogram, biochemistry, and histopathology

**DOI:** 10.1002/fsn3.4117

**Published:** 2024-06-12

**Authors:** Evan B. Craine, Mustafa Makav, Serpil Dağ, Ayfer Yıldız, Hüseyin Avni Eroğlu, Buket Boğa Kuru, Fikret Bektaşoğlu, Spencer Barriball, Brandon Schlautman, Muhammet Şakiroğlu

**Affiliations:** ^1^ The Land Institute Salina Kansas USA; ^2^ Department of Physiology, Faculty of Veterinary Medicine Kafkas University Kars Turkey; ^3^ Department of Pathology, Faculty of Veterinary Medicine Kafkas University Kars Turkey; ^4^ Department of Physiology, Faculty of Medicine Çanakkale Onsekiz Mart University Çanakkale Turkey; ^5^ Department of Animal Breeding and Husbandry, Faculty of Veterinary Medicine Kafkas University Kars Turkey; ^6^ Bioengineering Department Adana Alparslan Türkeş Science and Technology University Adana Turkey

**Keywords:** blood parameters, histopathology, Perennial Baki™ bean, rats, sainfoin seed

## Abstract

Sainfoin species (*Onobrychis* spp.) have been employed for centuries as an essential forage for ruminant animals, both for grazing and as hay. The seeds produced by sainfoin have also been investigated as an animal feed source and were indicated to be a particularly protein‐rich supplement for monogastric animals. This study explores the effects of two sainfoin seed inclusion rates in rat diets compared to a control diet, focusing on blood biochemical parameters and a comprehensive histopathological evaluation of multiple organ systems. Thus, we provide a novel contribution to the body of evidence investigating sainfoin seeds as a protein supplement in monogastric animal diets. In this 21‐day experiment, seven rats each were assigned to the control group, a 5% sainfoin seed group, and a 10% sainfoin seed group. The control group received standard feed and water; the second group received feed with 5% sainfoin seeds; and the third group received feed with 10% sainfoin seeds. At the experiment's end, necropsies and evaluations were conducted. Histopathological exams revealed normal organ structures in all 21 samples, regardless of the group. Blood analysis showed statistically significant decreases in creatine, ALT, P, Ca, and Mg levels in the sainfoin seed groups compared to the control group, with most values nearing reference levels, suggesting potential benefits. Notably, no adverse effects were observed when sainfoin seeds were included at 5% and 10% in the rat feed. These findings contribute to a growing body of research investigating the inclusion of sainfoin seeds in monogastric animal diets, which is a foundational component of assessing sainfoin's potential as a novel pulse crop for human consumption.

## Graphical abstract

The effects of two sainfoin seed inclusion rates in rat diets were compared to a control diet. Histopathological exams revealed normal organ structures in all 21 samples, regardless of the group. Blood analysis showed statistically significant decreases in creatine, ALT, P, Ca, and Mg levels in the sainfoin seed groups compared to the control group, with most values nearing reference levels, suggesting potential benefits. Notably, no adverse effects were observed when sainfoin seeds were included at 5% and 10% in the rat feed.

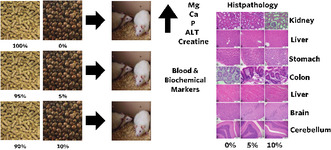

## 1 INTRODUCTION

Sainfoins (*Onobrychis* spp.) have been used in agriculture for centuries as perennial forage legumes and an important biological source of nitrogen (Hayot Carbonero et al., 2011). Sainfoins can enhance soil fertility through nitrogen fixation, animal nutrition by improving milk fatty acid profiles (Huyen et al., 2020; Menci et al., 2022), and the overall sustainability of agroecosystems in this role (Bhattarai et al., 2016; Sakhraoui et al., 2023). Additional benefits to soil conservation (e.g., reduced erosion) and health (e.g., composition, structure, and microbial activity) arise from their ability to provide continuous, multiyear living cover (Mora‐Ortiz & Smith, 2018; Nielsen, 2021). Sainfoins are also an excellent floral resource for honeybees and native bees (Kells, 2001; Kropacova & Haslbachova, 1970) and provide habitat for wildlife (USDA NRCS, 2013).

Their primary use has historically been for grazing and hay for ruminant animals. The unique phenolic profile of sainfoin foliage has been shown to have multiple benefits to ruminant health, mainly arising from proanthocyanidins (i.e., condensed tannins) (Sheppard et al., 2019; Waghorn & McNabb, 2003; Wang et al., 2015). These compounds can reduce *Escherichia coli* shedding in cattle compared to alfalfa (*Medicago sativa*) (Berard et al., 2009), reduce parasite loads as a natural anthelmintic (Desrues et al., 2016; Legendre et al., 2017), and reduce the risk of bloat often associated with other legume forages (Waghorn & McNabb, 2003). Specifically, the condensed tannins present in sainfoins reduce bloat and improve protein utilization by binding proteins and reducing nitrogen digestibility in the rumen (Kelln et al., 2021; Sheppard et al., 2019), and thus increase the flow of essential amino acids to, and absorption in, the small intestine (Herremans et al., 2020; Wang et al., 2015).

More recently, the seeds produced by sainfoin have also been investigated as a novel, sustainable perennial grain and animal feed source and have been shown to be a particularly protein‐rich supplement (Baldinger et al., 2016). The seeds and pods, or legumes, consist of approximately 28% protein, and protein content increases to approximately 38% when the pods are removed (Baldinger et al., 2016). Reports of sainfoin seed protein content range from 28.7% (Tarasenko et al., 2015) to 40.97% (Craine et al., 2023). Similarly, sainfoin seeds have been shown to have higher dietary fiber content than other legumes, with values similar to lupine (48.96%) (Craine et al., 2023). Overall, these findings demonstrate the potential for sainfoins to have comparable protein content to soybean (*Glycine max*) and lupines (*Lupinus* spp.), which are known as high protein sources, and higher protein content than most grain legumes (i.e., pulses).

Several studies have evaluated sainfoin seeds as a protein supplement in the diets of monogastric animals. Holden (1963) compared sainfoin seeds, soybean meal, and pigweed (*Amaranthus retroflexus*) and evaluated rat growth and feed consumption. They suggest that overall, sainfoin seeds compared favorably with soybean oil meal. Ditterline et al. (1977) compared sainfoin to soybean seed meal in two experiments with weanling rats and one experiment with weanling pigs, and quantified performance (e.g., average feed to gain ratio), trypsin inhibitor activity, impacts on pancreas health (i.e., size), and effects of processing such as autoclaving and oil extraction. Collectively, these experiments presented strong preliminary evidence for the safe use of sainfoin seeds in monogastric animal diets, as well as their viability as a feasible alternative to soybeans. Most recently, Baldinger et al. (2016) conducted a feeding trial to evaluate organic sainfoin seeds in the diets of weaned piglets. The trial included a control diet, two diets with 10% inclusion of sainfoin, with and without pods, and a diet with 16% inclusion of sainfoin seeds with pods. They concluded that an inclusion of 10%–16% sainfoin in weaned piglet diets can be a valuable addition, especially when sainfoin can be produced locally to offset expensive feeds such as imported, organic soybean seed meal. These studies support sainfoin seeds as an alternative to soybean seed meal in the diets of monogastric animals.

To build on these studies, our aim was to investigate the effects of 5% and 10% sainfoin seed inclusion rates in rat diets, in comparison to a control diet. Specifically, we performed a comprehensive evaluation of hematological and biochemical parameters, as well as a histopathological evaluation of multiple organ systems. Thus, we provide a novel contribution to the body of evidence investigating sainfoin seeds as a sustainable, alternative protein supplement in human and monogastric animal diets, using rats as a model. This study is part of ongoing research to evaluate sainfoin seeds as novel human and animal foods. Targeted domestication of sainfoins for this use is being led by The Land Institute with global partners. Perennial Baki™ bean refers to pulses, or grain legumes, derived from sainfoins. This process is comparable to Kernza®, the first commercially viable perennial grain crop, which has been in development since the early 1990s. The Land Institute owns and manages the Baki™ bean and Kernza® trademarks to ensure identity, food safety, and quality standards are met during the commercialization of these novel and emerging grain crops.

## 2 MATERIALS AND METHODS

### 2.1 Experimental design

The ethical approval for the present study was obtained from the Animal Experiments Local Ethics Board of Kafkas University (KAU‐HADYEK/2022–139) before the initiation of the study. A total of 21 4‐month‐old male Wistar albino rats were obtained and used in the current study. An equal number of seven rats were randomly assigned to three groups: control group, 5% sainfoin seed group, and 10% sainfoin seed group.

### 2.2 Feed preparation and analysis

Three different feed rations were prepared by a commercial company (Optima Feed, Bolu, Turkey). The routine pellet feed was used for the control groups (Table 1). Sainfoin seeds (cv. Delaney) were removed from the pod using a laboratory thresher and cleaner (Haldrup LT‐15, Ilshofen, Germany). Sainfoin seeds were not subjected to any heat treatment before grinding and were added to the routine pellet feed at 5% and 10% proportions to produce 5% and 10% sainfoin seed rations, respectively. The feed ingredients were ground and passed through a 2 mm sieve before being pelletized (Aksol Makina, Kahramankazan, Ankara, Turkey). The micronutrient and macronutrient composition of each of the feed treatments were determined using official methods of analysis (AOAC, 2003) and are provided in Table 2.

**TABLE 1:** Composition of routine pellet feed, as prepared and reported by the commercial company (Optima Feed, Bolu, Turkey).

Material	Minimum (%)	Maximum (%)
Soybean meal	36	40
Wheat bran	30	32
Corn	15	20
Rice bran	7	9
Molasses	4	5
Calcium carbonate	1	2
Dicalcium phosphate	1	2
Salt	0.5	1
Vegetable oil	0.5	1.5
Vitamin mineral premixes	0.2	0.5

*Note*: The content of each component is guaranteed to be within the ranges provided.

**TABLE 2:** Micronutrient and macronutrient composition of each of the feed treatments, including the routine pellet feed for rats (i.e., control), the 5% inclusion of ground sainfoin seeds (i.e., 5%), and the 10% inclusion of ground sainfoin seeds (i.e., 10%).

Content	Unit	Control diet	5% sainfoin seed	10% sainfoin seed
Calcium (Ca)	%	1.61	1.48	1.48
Magnesium (Mg)	%	0.40	0.38	0.41
Sodium (Na)	%	0.26	0.25	0.26
Phosphorous (P)	%	1.04	1.03	1.04
Potassium (K)	%	0.91	0.87	0.90
Iron (Fe)	mg/kg	204.9	233.6	226.8
Dry matter	%	95.21	94.80	94.99
Crude ash	%	8.84	8.62	8.32
Crude fat	%	5.43	5.00	4.92
Crude cellulose	%	5.48	6.10	5.80
Crude protein	%	23.08	23.38	23.42
Metabolic energy	kcal/kg	2890	2845	2871

### 2.3 Study duration, rat condition, and euthanasia

During the experiment, which lasted 21 days, routine pellet feed and drinking water were given to the control group, feed containing 5% sainfoin seeds to the second group, and feed containing 10% sainfoin seeds to the third group. At the end of the experiment period, systemic necropsies of all subjects were performed during euthanasia and follow‐up. The study was initiated on September 26, 2022 and concluded on October 17, 2022.

### 2.4 Hemogram and selected biochemical parameters

Blood samples were taken immediately after performing cervical vertebra dislocation per ethical rules on October 16, 2022. Blood and tissue samples were kept at −80°C until biochemical analyses. Biochemical analyses were performed by photometric methods using a commercial kit and Abbott Architect c8000 analyzer (Abbott Laboratories, an Abbott Park, IL, USA) and hemogram devices (VGMS4e®, Melet Schloesing, Fransa) at Kafkas University School of Veterinary Medicine Laboratory on October 22, 2022.

### 2.5 Pathological tests and procedures

Tissue samples (liver, kidney, stomach, colon, heart, brain, and cerebellum) taken after necropsy were examined macroscopically and microscopically. Tissue samples taken for microscopic examinations were subjected to routine tissue processing, and paraffin blocks were prepared. Sections of 5 μm thickness were taken from the paraffin blocks and examined under a light microscope. The presence of inflammatory cell infiltration in the removed organs was examined for detecting degenerative and necrotic changes. The tissues were photographed using an Olympus Bx53 camera and the Cell ^P program (Olympus Soft Imaging Solutions GmbH, 3.4).

### 2.6 Statistical analysis

Analysis of variance (ANOVA) was conducted for each variable, and mean separations among the groups for all variables were conducted using Tukey's multiple comparison test using inherent functions in the R program. Statistical significance was assessed at the 5% probability level, unless otherwise indicated.

## 3 RESULTS

### 3.1 Hemograms and selected biochemical parameters

A total of 46 blood parameters were measured and evaluated in the present study (Table 3). Of the 55 parameters, ANOVA analyses indicated that the group means were different in at least six variables (Table 3). We subsequently performed a Tukey's mean separation test to find the differences among groups.

**TABLE 3:** Hemogram and selected biochemical parameters in the present study, along with the significance of each parameter based on the one‐way ANOVA analyses.

Blood variable	Unit	Mean square	*F* value	*p* Value
White blood cells (WBC)	103/μL	10.23	1.87	.18
Red blood cell (RBC)	106/μL	1.38	3.11	.07
Hemoglobin (HGB)	g/dL	4.33	2.75	.09
Hematocrit (HCT)	%	16.14	2.73	.09
Mean corpuscular volume (MCV)	fL	1.76	0.60	.56
Mean corpuscular hemoglobin (MCH)	Pg	0.29	1.07	.37
Mean corpuscular hemoglobin concentration (MCHC)	g/dL	1.19	2.99	.08
Red blood cell distribution width (RDW)	%	0.24	0.15	.86
Platelet count (PLT)	103/μL	3002	0.36	.70
Mean platelet volume (MPV)	fL	0.32	2.96	.08
Lymphocyte (LYM)%	%	3.66	1.14	.34
Monocyte (MON)%	%	0.29	1.80	.20
Neutrophil (NEU)%	%	1.02	0.45	.64
Eosinophil (EOS)%	%	0.01	0.77	.48
Basophil (BAS)%	%	0.01	1.10	.36
Lymphocyte (LYM)	103/μL	7.93	1.67	.22
Monocyte (MON)	103/μL	0.004	3.22	.07
Neutrophil (NEU)	103/μL	0.13	2.28	.13
Eosinophil (EOS)	103/μL	0.0001	0.77	.48
Basophil (BAS)	103/μL	0.0001	1.04	.38
Glucose	mg/dL	3952	0.46	.64
Urea	mg/dL	32.30	2.14	.15
Creatinine	mg/dL	0.12	7.42	.004**
Triglyceride	mg/dL	389.1	2.52	.109
Cholesterol	mg/dL	108.55	2.86	.09
High‐density lipoprotein (HDL) cholesterol	mg/dL	45.79	3.03	.07
Low‐density lipoprotein (LDL) cholesterol	mg/dL	74.90	10.28	.001**
Aspartate transaminase (AST)	U/L	38.90	0.20	.82
Alanine aminotransferase (ALT)	U/L	1280	5.30	.02*
Gamma‐glutamyl transferase (GGT)	U/L	0.4	0.22	.80
Creatine kinase (CK)	U/L	64,587	2.25	.13
Direct bilirubin	mg/dL	0.00	0.99	.39
Total bilirubin	mg/dL	0.00	0.33	.72
Albumin	g/dL	7.04	2.71	.10
Lactate dehydrogenase (LDH)	U/L	90,345	2.41	.13
Total protein	g/dL	34.42	2.37	.12
Phosphorus (P)	mg/dL	16.61	8.20	.003**
Calcium (Ca)	mg/dL	5.57	7.72	.004**
Lipase	U/L	1.60	1.52	.25
Magnesium (Mg)	mg/dL	4.51	15.15	.0001***
Sodium (Na)	mEq/L	9.24	2.74	.10
Potassium (K)	mEq/L	2.53	2.75	.09
Iron (Fe)	mcg/dL	1047.9	2.78	.09
CRP	mg/dL	0.14	0.21	.81
Uric acid	mg/dL	28.19	1.72	.21
Amylase	U/L	905.3	3.52	.06

* *p* < .05.

** *p* < .01.

*** *p* = .001.

The hematological and biochemical parameters in the blood of rats with 5% and 10% sainfoin seeds added to the rations were compared with the rats that had no sainfoin seed in their diet (Table 2). The results indicated that a statistically significant decrease was present among the groups fed with sainfoin seeds compared to the control group for blood creatine, ALT, P, Ca, and Mg levels (Table 3). When looking at LDL‐C value, no significant difference was found between the control group and 5% group, whereas a significant decline was found in the 10% sainfoin group compared to the other two groups (Table 4).

**TABLE 4:** Means of the significant blood variables in three experimental groups (control, 5% sainfoin seed inclusion, and 10% sainfoin inclusion) along with the separation based on Tukey's multiple comparison test.

Groups	Creatine (mg/dL)	LDL C (mg/dL)	ALT (U/L)	P (mg/dL)	Ca (mg/dL)	Mg (mg/dL)
Control diet	0.71^a^	19.00^a^	69.29^a^	11.11^a^	11.67^a^	3.89^a^
5% sainfoin seed	0.54^b^	17.43^a^	44.57^b^	8.41^b^	10.43^b^	2.51^b^
10% sainfoin seed	0.52^b^	12.71^b^	47.43^b^	8.47^b^	9.93^b^	2.49^b^

*Note*: For each variable, values within rows followed by different letters are significantly different at *p* < .05.

### 3.2 Histopathological results

**FIGURE 1:**
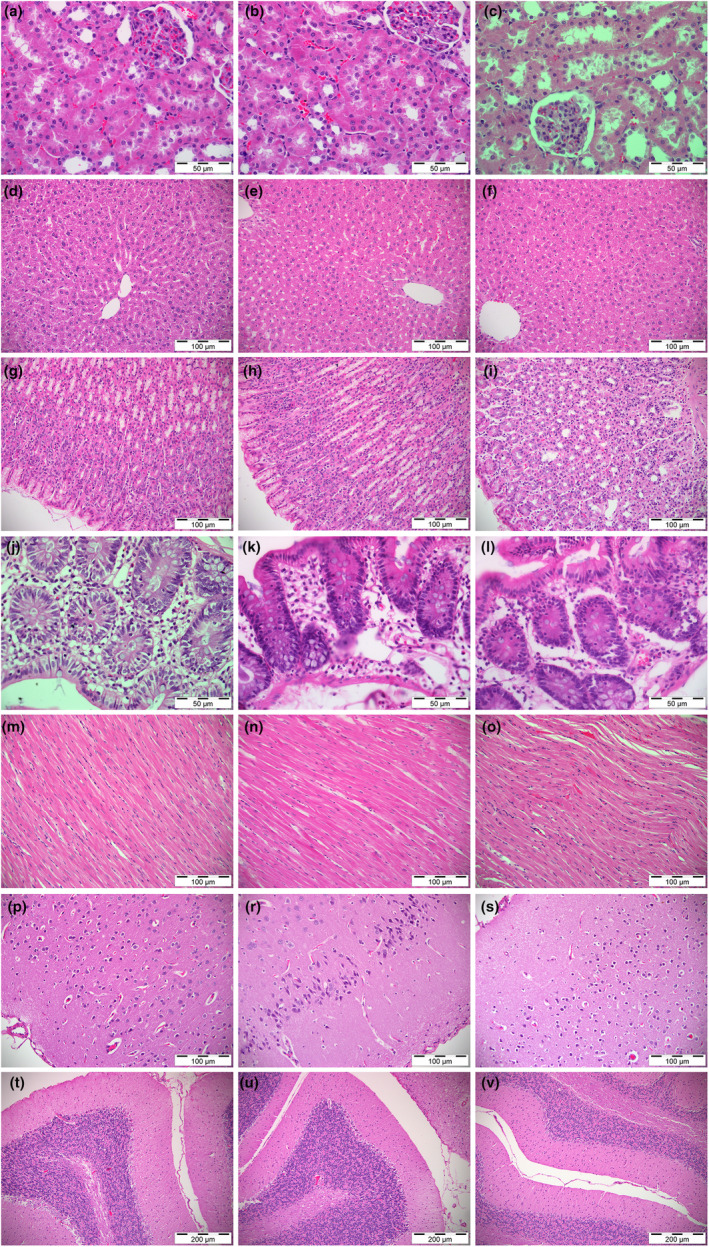
Histopathologic evaluations of the selected tissues of the three experimental groups. (a–c) kidney; (d–f) liver; (g–i) stomach; (j–l) colon; (m–o) heart; (p–s) brain; (t–v) cerebellum. The vertical columns are representing the groups.

The in‐depth analyses of histopathological examinations of tissue samples from the subjects indicated that all 21 samples were normal for all the organs analyzed. In other words, the samples from the group that were fed with 5% sainfoin seed feed (Figure 1B,E,H,K,N,R,U) and those that were in group 3 (10% sainfoin seed feed; Figure 1C,F,I,L,O,S,V) had a normal histological structure similar to the control group (Figure 1A,D,G,J,M,P,T). Animal tissues indicated no degenerative and necrotic changes. Nor did they exhibit any inflammatory infiltration pattern (Figure 1).

## 4 DISCUSSION

### 4.1 Diet treatment effects on hemogram, biochemistry, and histopathology

Our study examined the targeted effects of two different levels of sainfoin seed‐supplemented feed compared with the routine pellet feed. Ditterline et al. (1977) found raw sainfoin seeds to have relatively high trypsin inhibitor activity compared to sainfoin seeds autoclaved for 2 h, casein, and soybean meal. While heating raw sainfoin seeds effectively diminishes or trypsin inhibitor activity, rats fed raw sainfoin seeds did not exhibit diminished performance (defined pancreas enlargement) (Ditterline et al., 1977). This finding corroborated results from a separate experiment performed by Ditterline et al. (1977). Together, these studies indicate that it may not be necessary to inactivate the trypsin inhibitor before feeding raw sainfoin seeds. Therefore, sainfoin seeds were not subjected to any heat treatment before grinding.

Biochemical and histopathological evaluations were applied to blood and tissue samples. No difference between treatments was observed in the histopathology of a wide range of organs. Furthermore, a wide range of biochemical parameters were tested, and the obtained values were all within the reference ranges, confirming the histopathological findings. The absence of any pathological finding, and significantly positive differences in certain parameters, reveals that the inclusion of sainfoin seeds does not have any negative effect in rats and could be a reliable nutrient source.

Creatine, which is the breakdown product of creatine phosphate in muscle and protein metabolism, is released by the body at a constant rate proportional to muscle ratio (Lewis et al., 2014). Since serum creatinine is a byproduct of muscle metabolism that is continuously excreted by the kidneys and easily measured, it is an important indicator of kidney health. Intense exercise can increase creatinine by increasing muscle breakdown (Samra & Abcar, 2012). The reference range for rat creatinine levels is reported to be 0.2–0.8 mg/dL (Hrapkiewicz et al., 2013). Although critically low levels of creatinine in the blood could be attributed to malnutrition in the form of lower protein consumption (Hari et al., 2007; Ongan & Ersoy, 2012), here the blood creatinine levels of all the three groups are found to be within the reference range. The mean creatinine level of the control group was 0.71 mg/dL, which was closer to the upper end of the reference range. The value has significantly declined to 0.54 and 0.52 mg/dL for 5% and 10% sainfoin‐fed groups, respectively, indicating a decline toward the mean of the reference range.

The importance of lowering low‐density lipoprotein (LDL) cholesterol in the prevention of cardiovascular events is indicated. The level of LDL in the blood is associated with atherosclerosis and, therefore, coronary artery disease, stroke, and peripheral vascular diseases (Bayturan et al., 2011). At the same time, LDL cholesterol is also called malignant cholesterol. In our study, the decrease in LDL_C level in sainfoin groups is physiologically noteworthy.

The increase in ALT level specifically represents liver damage as well as adverse gastrointestinal related health events due to consumption of the food toxicants. In many studies, it has been observed that there is a significant increase in ALT level after liver damage (Adali et al., 2019; Eroğlu et al., 2020). A number of other reasons such as hemochromatosis, vascular disease, acute viral hepatitis, and genetic disorders were also cited for elevated ALT in blood serum (Moriles & Azer, 2022). Low levels of ALT in the blood is investigated largely in the context of mortality among elderly population (Ramaty et al., 2014).The reference range for ALT level in rats is reported to be 16–89 U/L (Hrapkiewicz et al., 2013). The blood ALT levels of the control group in this study as well as the sainfoin diet based groups are found to be in the reference range. The mean ALT level found among the control group was 69.29 U/L which was closer to the upper limit of the reference value. The value has declined to 44.57 and 47.43 U/L (closer to the mean of the reference range) for 5% and 10% sainfoin‐fed groups, respectively.

There was a decrease in P, Ca, and Mg parameters in the sainfoin groups compared to the control group. The observed decrease in blood P and Ca values of sainfoin‐fed groups could be interpreted as normalization toward the reference range reported in (Houtmeyers et al., 2016). In fact, the normal ranges for rats are reported to be between 2.3–7.0 mg/dL for phosphorus and 9.5–10.9 mg/dL for calcium. The Mg reference values are not well established in rats, and different studies have reported different reference values (Boguszewska‐Czubara, 2011; Hans et al., 2002; Watchorn, 1933). However, a similar normalization toward the reference range is also expected for Mg.

### 4.2 Sainfoin seeds as an alternative protein source for monogastric animal diets

In this study, no detrimental effects were observed in the rat biochemical, hematological and histopathological parameters measured in this study when sainfoin seeds were included at 5% and 10% of the rat feed ratio. These observations strengthen those from previous studies investigating the value of sainfoin as a feed source and protein supplement for monogastric animals by measuring growth parameters in rats and pigs (Baldinger et al., 2016; Ditterline et al., 1977; Holden, 1963).

Holden (1963) did not find any significant difference in weights of rats fed sainfoin seed or soybean meal, they note that rats fed sainfoin seeds consumed more feed and had lower feed efficiencies compared to rats fed soybean meal. Nevertheless, Holden (1963) suggests that sainfoin seeds compare favorably overall in comparison with soybean oil meal. Ditterline et al. (1977) performed two separate experiments with weanling rats to evaluate sainfoin seeds as a protein supplement. In the first experiment, sainfoin and soybeans were fed separately in the raw form, with oil extracted, or after being autoclaved in a diet consisting of 20% protein and 4% fat. Processing did not appreciably impact amino acid profiles of the six different protein sources. No significant differences were detected for average daily gain, protein efficiency ratios, and feed consumption between the diets. From data on a subset of rats fed each diet, that rats fed raw sainfoin had significantly lower pancreas weights compared to five other diets. This result indicated that the trypsin inhibitor present in sainfoin does not cause pancreas enlargement or decrease the feed value. Our results corroborate these findings. Histopathology did not reveal any pancreatic damage, and measures of blood glucose and amylase did not significantly differ between treatments. This is in contrast to the case with raw soybeans, where the trypsin inhibitor present must be inactivated before feeding. In the second experiment performed by Ditterline et al. (1977), the rat diets were adjusted to 11% protein content. No significant differences were detected for average daily gain between rats fed raw sainfoin, soybean meal, or casein. Furthermore, no significant difference was observed for the average feed to gain ratio raw sainfoin and soybean meal. Rats fed soybean meal had better feed efficiency than sainfoin, and no differences were observed between the sainfoin diets. With the results of the first experiment, these results indicate that rats generally performed equally well on sainfoin and soybean meal.

Prior to a wheeling pig feeding study, Ditterline et al. (1977) determined protein content and found sainfoin seeds with oil extracted (36.5%) and without oil extracted (35.9%) to have lower protein content than the soybean meal (50.4%) evaluated. However, the amino acid profiles were found to be quite similar, indicating that the protein sources should satisfy the essential amino acid requirements of weanling pigs. While Ditterline et al. (1977) found no significant differences between rations for average daily gain, total gain, daily feed consumed, and feed per gain ratios, it was suggested that pigs fed the soybean meal diet performed better, although the trial length was deemed insufficient to provide definitive evidence. Baldinger et al. (2016) found no difference in weight gain for weaned piglets with up to 16% inclusion of sainfoin. They note the potential value of sainfoin seeds as a substitute for peas or soybean, due to the favorable ratio of amino acids observed, especially the content of lysine, methionine and cysteine, threonine, and tryptophan.

The sainfoin fruit, or legume, consists of approximately 40% pod and 60% seed by weight, with the pods consisting of approximately 10%–11% protein, 1%–4% fat, 7%–10% ash, 4%–10% water‐soluble carbohydrates, and 8%–17% lignin (Craine & Schlautman, 2024). Woodman and Evans (1947) fed sainfoin with and without pods in a digestion trial with two wether sheep. They found that including the pods increased the lime and fiber content and slightly decreased the protein content of the feed. In their study, they suggest that removing the pods would produce a feed of similar quality in terms of digestion coefficients to the alfalfa and clover seed meals also evaluated. Baldinger et al. (2016) did not observe any differences when sainfoin seeds were fed with and without the seed pods. Therefore, feeding sainfoin with or without the pods seems to present viable options, depending on considerations of any constraints related to processing to remove the pods, or transportation of the feed without the pod fraction to concentrate the protein content and reduce shipping costs.

With corroborating past research, this study indicates that ongoing research about the potential use of sainfoin seeds as an alternative protein supplement for human and/or monogastric animal diets is justified. Through a comprehensive histopathological, hematological, and biochemical evaluation, this study contributes to a growing body of evidence demonstrating the potential of sainfoin as a novel, perennial pulse crop, which academic and non‐profit researchers internationally are working to develop under the name Perennial Baki™ bean. However, ongoing research is still needed to continue building the body of evidence necessary for scalable commercialization and adoption of sainfoin seeds in human and animal foods. For example, bioactive compounds, including tannins, have been observed in sainfoin seeds (Craine et al., 2023; Goplen et al., 1980; Lees et al., 1993; Wijekoon et al., 2021). Although we did not observe negative effects of sainfoin on measured parameters in this study, nor were they observed for growth parameters in previous studies previously shown for weight gain in a piglet feeding study (Baldinger et al., 2016), research investigating the positive or negative effects of tannins and other potentially bioactive compounds present in sainfoin seeds should merit additional research as the seeds are considered in human and animal feed diets.

## AUTHOR CONTRIBUTIONS

**Evan B. Craine:** Investigation (supporting); methodology (supporting); resources (equal); software (equal); writing – original draft (equal); writing – review and editing (equal). **Mustafa Makav:** Data curation (equal); investigation (equal); methodology (equal); resources (equal); software (equal); validation (equal); writing – original draft (equal); writing – review and editing (equal). **Serpil Dağ:** Investigation (equal); resources (equal); software (equal); validation (equal); visualization (equal); writing – review and editing (equal). **Ayfer Yıldız:** Data curation (equal); resources (equal); software (equal); writing – review and editing (equal). **Hüseyin Avni Eroğlu:** Data curation (equal); resources (equal); software (equal); writing – review and editing (equal). **Buket Boğa Kuru:** Data curation (equal); methodology (equal); resources (equal); software (equal); validation (equal); visualization (equal); writing – review and editing (equal). **Fikret Bektaşoğlu:** Data curation (equal); resources (equal); software (equal); writing – review and editing (equal). **Spencer Barriball:** Methodology (equal); resources (equal); software (equal); writing – review and editing (equal). **Brandon Schlautman:** Conceptualization (equal); investigation (equal); methodology (equal); project administration (equal); resources (equal); software (equal); supervision (equal); validation (equal); writing – original draft (equal); writing – review and editing (equal). **Muhammet Şakiroğlu:** Conceptualization (equal); formal analysis (lead); funding acquisition (lead); investigation (equal); methodology (equal); project administration (equal); resources (equal); software (equal); supervision (equal); validation (equal); visualization (equal); writing – original draft (equal); writing – review and editing (equal).

## FUNDING INFORMATION

This study was supported by Scientific and Technological Research Council of Turkey (TUBITAK) under the Grant Number 2247‐120C136. The authors thank to TUBITAK for their supports.

## CONFLICT OF INTEREST STATEMENT

Certain authors are affiliated with The Land Institute, which owns the trademark perennial Baki™ bean. The Land Institute is a 501(c)(3) non‐profit organization based in Salina, Kansas, that was founded in 1976. The Land Institute co‐leads the global movement to develop perennial grains, pulses, and oilseed‐bearing plants to be grown in ecologically intensified, diverse crop mixtures known as perennial polycultures. Kernza® perennial grain is being domesticated from intermediate wheatgrass as a grain for human food, and Perennial Baki™ bean is being domesticated from sainfoins as a pulse crop. The trade names are registered and employed to ensure quality oversight for the emerging perennial grain crops.

## Data Availability

Data are available by request at Sainfoin Consortium Germinate https://sainfoinconsortium.org/#/home.
